# CDKI-73 Is a Novel Pharmacological Inhibitor of Rab11 Cargo Delivery and Innate Immune Secretion

**DOI:** 10.3390/cells9020372

**Published:** 2020-02-05

**Authors:** Alexandra Sorvina, Tetyana Shandala, Shudong Wang, David J. Sharkey, Emma Parkinson-Lawrence, Stavros Selemidis, Douglas A. Brooks

**Affiliations:** 1 Cell Biology and Disease Research Group, Cancer Research Institute, University of South Australia, Adelaide, SA 5000, AustraliaEmma.Parkinson-Lawrence@unisa.edu.au (E.P.-L.); 2 Centre for Drug Discovery and Development, Cancer Research Institute, University of South Australia, Adelaide, SA 5000, Australia; Shudong.Wang@unisa.edu.au; 3 Robinson Research Institute, University of Adelaide, Adelaide, SA 5000, Australia; David.Sharkey@adelaide.edu.au; 4 School of Health and Biomedical Sciences, RMIT University, Bundoora, VIC 3083, Australia; Stavros.Selemidis@rmit.edu.au

**Keywords:** CDKI-73, endosomes, Rab11, antimicrobial peptide Drosomycin, IL-6, TNFα, *Drosophila*

## Abstract

Innate immunity is critical for host defence against pathogen and environmental challenge and this involves the production and secretion of immune mediators, such as antimicrobial peptides and pro-inflammatory cytokines. However, when dysregulated, innate immunity can contribute to multifactorial diseases, including inflammatory rheumatic disorders, type 2 diabetes, cancer, neurodegenerative and cardiovascular diseases and even septic shock. During an innate immune response, antimicrobial peptides and cytokines are trafficked via Rab11 multivesicular endosomes, and then sorted into Rab11 vesicles for traffic to the plasma membrane and secretion. In this study, a cyclin-dependent kinase inhibitor CDKI-73 was used to determine its effect on the innate immune response, based on previously identified targets for this compound. Our results showed that CDKI-73 inhibited the delivery of Rab11 vesicles to the plasma membrane, resulting in the accumulation of large multivesicular Rab11 endosomes near the cell periphery. In addition to the effect on endosome delivery, CDKI-73 down-regulated the amount of innate immune cargo, including the antimicrobial peptide Drosomycin and pro-inflammatory cytokines interleukin-6 (IL-6) and tumour necrosis factor alpha (TNFα). We concluded that CDKI-73 has the potential to regulate the delivery and secretion of certain innate immune cargo, which could be used to control inflammation.

## 1. Introduction

Innate immunity is the first line of host defence against pathogenic challenge and relies on immune cell recognition systems that trigger the production and secretion of immune mediators. If unchecked, the secretion of immune mediators can result in chronic inflammation and this is an important issue for patients with rheumatoid arthritis, type 2 diabetes, cancer and cardiovascular disease [1,2,3]. Inflammatory disorders represent a significant burden to both patients and health care systems, justifying the development of new therapeutics that can control the innate immune system.

The secretion of immune mediators by innate immune cells, such as macrophages, granulocytes and mast cells, involves the stepwise delivery of this immune cargo from biosynthetic compartments through sorting endosomes into specialist secretory vesicles. For example, the newly synthesised pro-inflammatory cytokines, interleukin-6 (IL-6) and tumour necrosis factor alpha (TNFα), are delivered as individual or mixed cargo in post-Golgi vesicles to recycling endosomes for sorting and packaging into specialist vesicles that are transported to the cell surface for secretion [4,5]. In RAW264.7 murine macrophages, membrane-bound TNFα and soluble IL-6 exit independently from recycling endosomes and are distinctly targeted to the cell surface [4]. Consequently, while TNFα is trafficked towards the cell surface at the site of phagocytic cups [6], the transport of IL-6 is excluded from movement to this site [4], but is still delivered to the cell surface. Rab GTPases are critically involved in the regulation of this sequential transport process, with for example, cargo sorting in Rab4/Rab11 recycling endosomes [7] and traffic to the plasma membrane in small Rab11 vesicles [8]. Rab11-mediated exocytosis has been reported for various cytokines and antimicrobial peptides, including TNFα [6,9], interferon gamma [9], IL-10 in mammals [5] and the antimicrobial peptide Drosomycin (Drs) in *Drosophila* [8]. These Rab4/Rab11-dependent sorting and Rab11-dependent exocytosis pathways represent potential targets for the development of new therapeutics to control innate immune secretion.

The activity of Rab11 is directly controlled by guanine nucleotide exchange factors (GEFs), which mediate the exchange from guanosine diphosphate to guanosine triphosphate [10,11], and GTPase activating proteins (GAPs), which facilitate GTPase activation by catalysing the dephosphorylation of guanosine triphosphate to guanosine diphosphate [12,13]. For example, *Drosophila* Crag or calmodulin-binding protein related to a Rab3 GDP/GTP exchange protein [14], human dual-specific A-kinase-anchoring protein 2 (D-AKAP2, also known as AKAP10) [15] and *Drosophila* Pkaap [16] appear to have GEF activity towards Rab11. Three Rab11 GTPase activating proteins have been identified, including the *Drosophila* protein Evi5 [17,18] and domain proteins TBC1D11/GAPCenA [19] and TBC1D15 [20]. Rab11 activity during vesicle trafficking can also be regulated by the *Drosophila* Lyst, also known as Blue cheese [21]. Functional defects in human and mouse LYST (also known as Chédiak-Higashi/Beige) result in the appearance of large lysosome-related compartments with impaired secretion and increased susceptibility to infection, while loss of *LYST* gene causes a severe immunodeficiency such as Chédiak-Higashi syndrome [22]. Interestingly, depletion of *LYST* in human epithelial cells has shown no effect on trafficking of endocytic cargo via retrograde transport, endocytic degradation or autophagy [23]. The modulation of immune cargo exocytosis and secretion, by targeting these Rab11 regulatory proteins is an avenue for the development of new therapeutics to control inflammatory diseases.

Cyclin dependent kinases are involved in the control of transcription for multiple genes [24], and are potential candidates for the regulation of Rab11 vesicle sorting and the secretion of innate immune cargo. Here, a specific focus on CDKI-73 (12e) [25], a derivative of *n*-phenyl-4-(thiazol-5-yl)pyrimidin-2-amine, was employed based on previously identified targets for this compound, including CDK9 and eukaryotic translation initiation factor 4E (eIF4E) [26], which control the immune response and inflammation. The primary screens examining the efficiency of CDKI-73 in controlling an innate immune response were achieved in the fly *Drosophila melanogaster*, which has been incorporated into the therapeutic discovery process for human inflammatory disorders [27]. Unlike mammals, the *Drosophila* immune system only exhibits innate immune function. It is mainly mediated by the fat body, the cells of which are large (high DNA ploidy), with proportionally enlarged intracellular compartments, and haemocytes (professional macrophages). This provided an ideal system to study the effect of CDKI-73 on endosomes during an innate immune response. Our results revealed that CDKI-73 prevented the delivery of Rab11 vesicles to the plasma membrane, resulting in the accumulation of large multivesicular Rab11 endosomes at the cell periphery, and effectively this decreased the level of antimicrobial peptide Drs and pro-inflammatory cytokine secretion. This effect on innate immune cargo delivery and secretion was demonstrated in both *Drosophila* and mammalian macrophages.

## 2. Materials and Methods

### 2.1. Fly Stocks

Fly stocks were maintained in standard medium at 25 °C [8]. The yeast *GAL4-UAS* system was used for fat body-specific gene expression [28] and transgene expression was driven by *CG-GAL4* [29]. *UAS-Rab11-GFP* transgenic stock was obtained from Markos González-Gaitán (University of Geneva, Geneva, Switzerland) and Donald F. Ready (Purdue University, West Lafayette, IN, USA). *UAS-lyst^RNAi^* transgenic stock was obtained from the Bloomington *Drosophila* Stock Centre (Indiana University, Bloomington, IN, USA). Note that *Drosophila lyst* orthologue used in this study is blue cheese (*bchs*).

### 2.2. Natural Bacterial Infection of Drosophila Larvae

Early third instar *Drosophila* larvae were infected orally with *Micrococcus luteus* in 5% sucrose (OD_600_ ~ 200) for 105 min at 25 °C, avoiding temperature stress [8]. Control non-infected larvae were nurtured with sterile-filtered 5% sucrose for an equal time period.

### 2.3. Drug Treatment of the Fat Body Tissues

The stock solutions of 3-(5-fluoro-4-(4-methyl-2-(methylamino)thiazol-5-yl)pyrimidin-2-ylamino)benzenesulfonamide (CDKI-73) [25] and 5,6-dichloro-1-β-d-ribofuranosylbenzimidazole (DRB) compounds [30] were prepared at 10 mM in DMSO (#D2650, Sigma-Aldrich, St. Louis, MO, USA), which were diluted in sterile phosphate buffered saline (PBS; #D8537, Sigma-Aldrich, St. Louis, MO, USA) to a final concentration of 50 nM, 500 nM and 1 μM for CDKI-73 and 100 μM for DRB. The fat body tissues from late third larval instars (−4 h before puparium formation) were dissected into sterile PBS, and then transferred to an Eppendorf tube containing 300 µL of either PBS (controls), CDKI-73 at 50 nM, 500 nM and 1 μM or DRB at 100 μM [30]. To analyse the morphological changes in Rab11 endosomes, the fat body tissues were treated with CDKI-73 and DRB for 15 min at room temperature and then imaged at 15, 30 and 45 min.

To analyse the effect of CDKI-73 on antimicrobial peptide *Drs* gene expression by quantitative real-time PCR analysis (qRT-PCR), fat body tissues from infected larvae were incubated in either PBS (controls) or CDKI-73 compound (500 nM and1 μM) for 30 min at room temperature and then rinsed in PBS. The fat body tissues were collected, snap frozen on dry ice and stored at −80 °C for qRT-PCR. To determine the inhibitory effect of CDKI-73 treatment on innate immune cargo secretion (Drs-GFP), fat body tissues from infected late third larval instars were dissected and then treated for 30 min at room temperature with either PBS (controls), or CDKI-73 at 500 nM or 1 μM. The imaging of these tissues was done within 45 min.

The effect of CDKI-73 treatment on secretion was evaluated in fat body tissues, but not in hemolymph. The quantification of secreted protein into the hemolymph wasnot technically possible due to the extensive coagulation of the insect hemolymph upon exposure to air, making this fluid non-homogeneous and not suitable for quantification by spectrofluorometry [31,32].

### 2.4. Drs Gene Expression Analysis

For qRT-PCR analysis, RNA was isolated from the fat body tissue of 30 larvae, using a PureLink^®^ RNA Mini Kit (#12183018A and #12185010, Ambion, Austin, TX, USA). cDNA was synthesised using a High Capacity RNA-to-cDNA kit (#4387406, Applied Biosystems, Waltham, MA, USA). qRT-PCR was performed using Fast SYBR^®^ Green Master Mix kit (#4385616, Applied Biosystems, Waltham, MA, USA) and a 7500 Fast Real-Time PCR System (Applied Biosystems, Waltham, MA, USA). Three independent biological samples were analysed for each treatment group; each biological sample contained fat body tissues collected from 30 larvae. The mRNA expression of genes was normalised against the endogenous control gene *rp49* using the ΔΔCT method. PCR primers were obtained from GeneWorks (Adelaide, Australia). The primers used for the qRT-PCR included: *Drs* (CG10810) forward, 5′-GTACTTGTTCGCCCTCTTCG-3′, and reverse, 5′-ATTTAGCATCCTTCGCACCA-3′; and *rp49* (CG7939, used as an endogenous control) forward, 5′-CGAGTTGAACTGCCTTCAAGATGACCA-3′, reverse 5′-GCTTGGTGCGCTTCTTCACGATCT-3′.

### 2.5. Live Cell Imaging of the Fat Body Tissues

For ex vivo live cell imaging experiments, the fat body tissues were stained with the plasma membrane stain CellMask™ Deep Red (#C10046, Invitrogen, Waltham, MA USA), and then attached to a coverslip using Carbomer 940 based gel (Snowdrift Farm, Teton Valley, ID, USA) [33]. The ex vivo analysis of fat body tissues was limited to 45 min to preserve tissue integrity [33]. Imaging analysis was performed using a Zeiss LSM710 NLO confocal microscope, equipped with Argon-gas and 633 nm solid-state lasers (Zeiss, Germany). Each confocal micrograph represented 1.5 μm thin optical sections. Image processing was conducted with Adobe Photoshop CS6 (Adobe Systems Inc., San Jose, CA, USA).

### 2.6. Fat Body Viability Assay

Cellular NAD(P)H-dependent redox activity was measured using CellTiter 96^®^ AQueous Non-Radioactive Cell Proliferation Assay (MTS) according to the manufacturer’s instruction (#K300-500, Sapphire Bioscience, Redfern, Australia). Fat body tissues were dissected in PBS from *CG-GAL4 > UAS-Rab11-GFP/+* transgenic flies and incubated with either PBS or CDKI-73 compound for 15 min and then transferred to 120 μL of 3-(4,5-dimethylthiazol-2-yl)-5-(3-carboxymethoxyphenyl)-2-(4-sulfophenyl)-2H-tetrazolium and phenazine methosulfate in DMEM medium (#D6171, Sigma-Aldrich, St. Louis, MO, USA) in a 96-well plate. The tissues were allowed to incubate at room temperature for five hours and the absorbance was then measured at 490 nm using a VICTOR X™ Multilabel Plate Reader (PerkinElmer, Waltham, MA, USA). Data represents the mean ± SEM of three biological replicates for each group.

### 2.7. Cell Culture

Human monocytic leukemia THP-1 cells were obtained from the American Type Culture Collection (Sigma-Aldrich, St. Louis, MO, USA) and maintained in RPMI-1640 medium (#R0883, Sigma-Aldrich, St. Louis, MO, USA), containing 10% fetal bovine serum (#IVT3008403, In Vitro Technologies, Auckland, Australia) and 2 mM L-glutamine (#25030-081, Gibco, Waltham, MA, USA) at 37 °C and 5% CO_2_ in a Sanyo MCO-17AI humidified incubator (Sanyo Electric Biomedical Co., Ltd., Osaka, Japan). The THP-1 cells were cultured in 75 mm^2^ flasks, and cells (1 × 10^6^ cells/mL) that had been passaged from two up to five times were used for the experiments. THP-1 monocytes were differentiated into macrophages in 6-well plates by incubation with 3 mL of RPMI-1640 medium, containing 5 ng/mL of phorbol 12-myristate 13-acetate (#P8139, Sigma-Aldrich, St. Louis, MO, USA) over 48 h [34]. For lipopolysaccharide (LPS) treatment, the macrophages were washed with PBS and pre-treated with 100 ng/mL of LPS from *Escherichia coli* 0111: B4 (#L3024, Sigma-Aldrich, St. Louis, MO, USA) in 2 mL of serum free RPMI-1640 medium for two hours at 37 °C and 5% CO_2_. The supernatants were aspirated and the macrophages treated with either 100 ng/mL of LPS (controls) or 100 ng/mL of LPS together with different concentrations of CDKI-73 (50 nM and 100 nM) for four hours at 37 °C. The supernatants were collected and stored at −80 °C ready for assaying by ELISA.

### 2.8. ELISA

The amount of IL-1β, IL-6, IL-8 and TNFα in culture supernatants were determined using an Inflammatory Cytokine Human 5-Plex Panel for the Luminex^®^ platform, according to the manufacturers instruction (#LHC0003, Novex^®^, USA). The fluorescence was determined by Luminex xMAP^®^ technology (Millipore, Billerica, MA, USA). Data represents the mean ± SEM of three biological replicates for each group.

### 2.9. Immunostaining and Confocal Microscopy

The THP-1 macrophages were fixed and stained according to a previously described protocol [8]. Antibodies for immunofluorescence were mouse monoclonal anti-Rab11 (#610657, BD Transduction Laboratories™, USA), rabbit polyclonal anti-TNFα (#ab6671, Sapphire Bioscience, Redfern, Australia) and rabbit polyclonal anti-IL-6 (#ab6672, Sapphire Bioscience, Redfern, Australia). Secondary anti-IgG antibody conjugates with Cy5 labels were obtained from Jackson Immuno Research Laboratories. The Alexa Fluor^®^ 488 Phalloidin (#A12379) and Hoechst 33,258 DNA (#H3569) stains were obtained from Invitrogen, USA. Cell biology analysis was carried out with a minimum of three biological samples. Imaging analysis was performed using a Zeiss LSM710 NLO confocal microscope, equipped with Argon-gas and 633 nm solid-state lasers (Zeiss, Germany) and a two-photon Mai-Tai^®^ tunable Ti:Sapphire femtosecond pulse laser (Spectra-Physics, St. Louis, MO, USA). Each confocal micrograph represented 1.5 μm thin optical sections. Image processing was conducted with Adobe Photoshop CS6 (Adobe Systems Inc., San Jose, CA, USA).

### 2.10. Cell Viability Assay

The metabolic activity of THP-1 macrophages was measured using a Resazurin based In Vitro Toxicology Assay Kit (#TOX-8), according to the manufacturer’s instructions (Sigma-Aldrich, USA). THP-1 macrophages were seeded in 96-well plates at a density of 6.8 × 10^5^ cells/mL in the absence or presence of CDKI-73 compound at final concentrations of 50 nM, 100 nM, 500 nM, 1 µM, 10 µM, 50 µM and 100 µM. The Resazurin dye solution was added in an amount equal to 10% of the culture medium volume. The macrophages were then incubated for four hours at 37 °C under 5% CO_2_. The fluorescence of resofurin produced by macrophages was measured using an EnVision multi-label plate reader (PerkinElmer, Beaconsfield, UK) at an excitation wavelength of 560 nm and an emission wavelength of 590 nm. Data represents the mean ± SEM of four biological replicates for each group.

### 2.11. Measurements and Statistical Analysis

In *Drosophila* fat body cells, the number of small (≤1 µm) Rab11 vesicles at the plasma membrane (30 µm sections) and the size of multivesicular Rab11 endosomes throughout the cells (within 40 µm^2^ regions of interest (ROI)) were defined using Volocity 6.2.1 software (PerkinElmer, USA). Note that the size of small Rab11 vesicles was close to the resolution limit of the microscope, and therefore the slight variations in their morphology could not be precisely determined. The fluorescence intensity of Drs-GFP in fat body cells (within 50 µm^2^ ROI) and at the plasma membrane (15 µm x 3 µm ROI) were also measured using Volocity 6.2.1 software. The total fluorescence intensity was corrected by subtracting background intensity from the one measured in the ROI. In THP-1 macrophages, the fluorescence intensity of Cy5 was determined within the cells. The difference between group means was assessed by one-way analysis of variance (ANOVA), with individual group variance assessed by a Bartlett’s test. Where the level of significance was *p* < 0.05, post-hoc tests were performed using a Tukey’s multiple comparison test (using GraphPad Prism version 6.00 for Windows, GraphPad Software, San Diego, CA, USA). Data was presented as the mean ± SEM.

## 3. Results

### 3.1. CDKI-73 Altered the Morphology of Large Rab11 Endosomes

**Figure 1:**
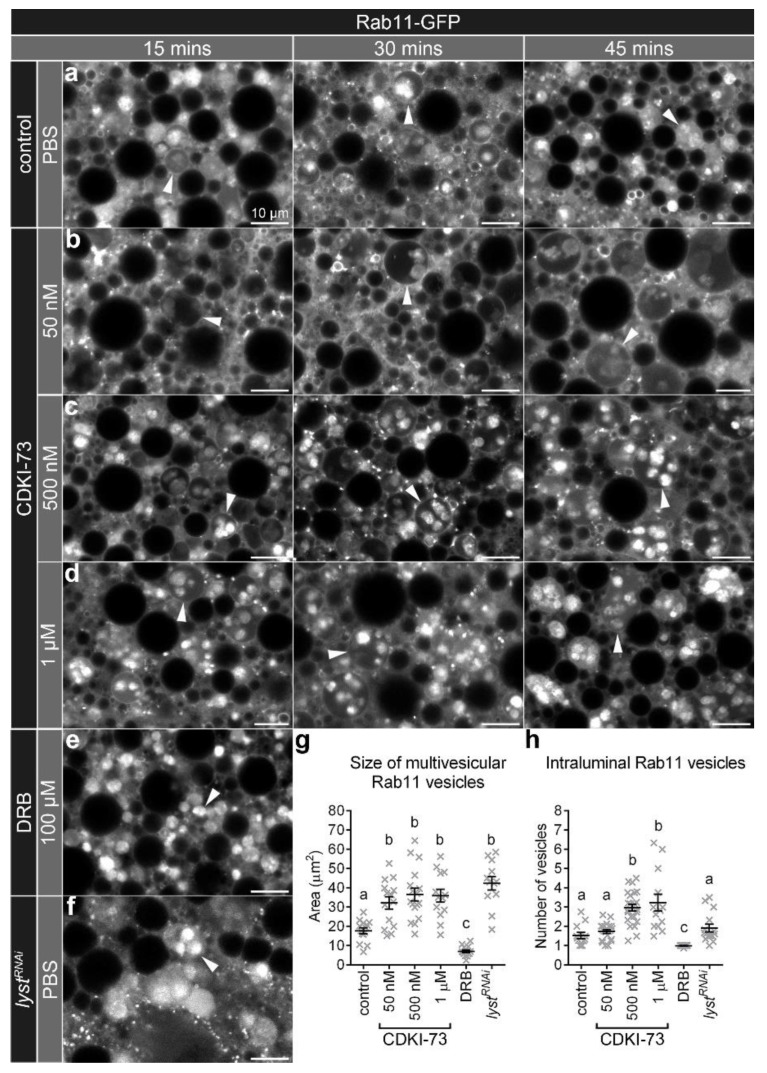
CDKI-73 treatment alters morphology of Rab11 endosomes. (**a**) Confocal micrographs of cross-sections through fat body cells showing Rab11-GFP endosomes. Representative images were from fat body tissues treated either with PBS (**a**,**f**), CDKI-73 at 50 nM (**b**), 500 nM (**c**), 1 μM (**d**) or DRBat 100 μM (**e**). Fat body cells were from the following genotypes: *CG-CAL4 > UAS-Rab11-GFP/+* (**a**–**e**) and *UAS-lyst^RNAi^/+; CG-CAL4 > UAS-Rab11-GFP/+* (**f**). Arrowheads depict large multivesicular Rab11 endosomes. Scale bars: 10 μm. Histograms showing comparative analysis of the size of multivesicular Rab11 endosomes (**g**), and the number of Rab11 intraluminal vesicles per multivesicular Rab11 endosome (**h**). One-way ANOVA and Tukey’s multiple comparison test showed significant differences between the means in designated groups (depicted by different letters on the bars, *p* < 0.0001). Data are represented as mean ± SEM.

In control *Drosophila* fat body cells, Rab11 endosomes were observed as either small vesicles or larger endosomes that contained Rab11 intraluminal vesicles (multivesicular endosomes; Figure 1a). The treatment of fat body cells with CDKI-73 did not appear to change the morphology of the small Rab11 vesicles (≤1 µm; Figure 1a–d), but resulted in a significant increase in their numbers, when compared to the controls (*p* < 0.05; Figure S1a). Notably, the size of Rab11 multivesicular endosomes in fat body cells was significantly increased after CDKI-73 treatment (Figure 1a–d,g; *p* < 0.0001). This effect on the size of the Rab11 multivesicular endosomes was evident at different doses of CDKI-73 (50 nM, 500 nM and 1 µM; Figure 1b–d), with slight differences observed within 30 and 60 min post-treatment (Figure S1b). As there were no significant differences at each of the time points after incubation, the data was plotted together representing the overall effect of CDKI-73 treatment on multivesicular endosomes (Figure 1g,h). The number of Rab11 intraluminal vesicles in these multivesicular endosomes was significantly increased with 500 nM and 1 µM CDKI-73 treatment, when compared to the untreated controls (*p* < 0.0001; Figure 1h). Treatment with 100 µM DRB, a highly selective CDK9 inhibitor, did not produce the same effect on Rab11 multivesicular endosomes (Figure 1e,g). However, RNAi-mediated silencing of the lysosomal trafficking regulator gene (*lyst*) produced an increase in the number of small Rab11 vesicles (≤1 µm) as well as size of Rab11 multivesicular endosomes (Figure 1f), which was comparable to CDKI-73 treatment (Figure 1g, Figure S1a). *lyst^RNAi^* did not result in a significant increase in the number of Rab11 intralumenal vesicles, when compared to the controls, and was therefore distinct from the response to 500 nM and 1 µM CDKI-73 treatments (Figure 1h). The morphological changes to Rab11 multivesicular endosomes did not appear to be due to cytotoxic effects, as the viability of fat body cells treated with CDKI-73 (50 nM–1 µM) was not significantly different from controls (Figure S2a). However, at the higher concentration of 10 mM, CDKI-73 treatment significantly decreased the viability of *Drosophila* fat body cells (*p* = 0.03; Figure S2b).

### 3.2. CDKI-73 Increased Rab11 Tubule Formation and Reduced Rab11 Vesicle Delivery to the Plasma Membrane

**Figure 2:**
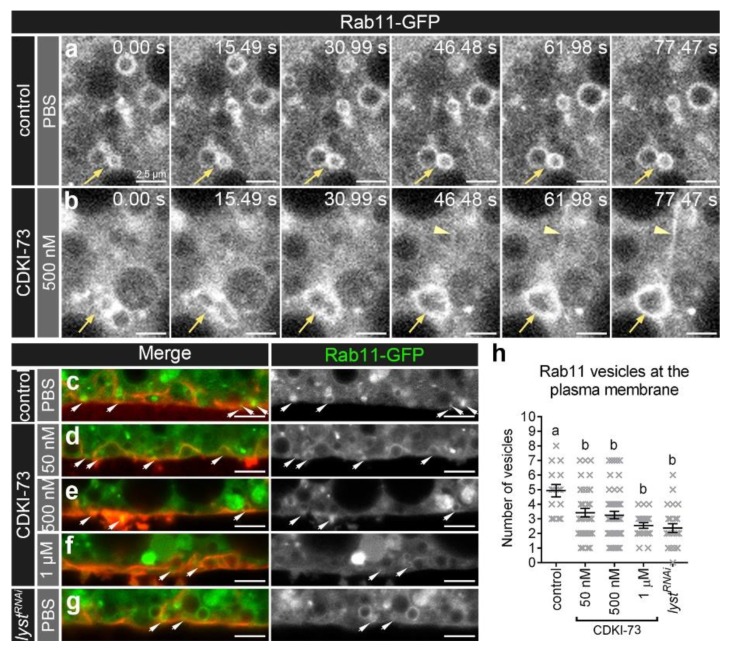
CDKI-73 induces homotypic vesicle fusion and reduces number of Rab11 vesicles at the plasma membrane. (**a**,**b**) Time-lapse confocal imaging of cross-sections through fat body cells showing Rab11-GFP endosomes. Representative images were from fat body tissues (*CG-CAL4 > UAS-Rab11-GFP/+*) treated either with PBS (**a**) or 500 nM CDKI-73 (**b**). The acquisition speed was set to 0.3 frames/second. Arrows depict fusing Rab11 endosomes. Arrowheads show Rab11 tubular structures. Scale bars: 2.5 μm. (**c**–**g**) Confocal micrographs of cross-sections through the fat body cells showing Rab11-GFP vesicles (green) in relation to the plasma membrane outlined by CellMask™ Deep Red (red). Representative images were from fat body tissues treated either with PBS (**c**,**g**) or CDKI-73 at 50 nM (**d**), 500 nM (**e**) and 1 μM (**f**). Fat body tissues were from the following genotypes: *CG-CAL4 > UAS-Rab11-GFP/+* (**c**–**f**) and *UAS-lyst^RNAi^/+; CG-CAL4 > UAS-Rab11-GFP/+* (**g**). Arrows depict small ≤1 µm Rab11 vesicles at the plasma membrane. Scale bars: 5 μm. (**h**) Histogram showing comparative analysis of the number of small ≤1 µm Rab11 vesicles at the plasma membrane. One-way ANOVA and Tukey’s multiple comparison test showed significant differences between the means in designated groups (depicted by different letters on the bars, *p* < 0.0001). Data are represented as mean ± SEM.

In fat body cells treated with CDKI-73 (500 nM), there were frequent fusion events observed for Rab11 endosomes (Figure 2b), but these events were less evident in control fat body cells (Figure 2a). These fusion events were not observed in *lyst^RNAi^* depleted fat body cells (data not shown). In addition, CDKI-73 caused Rab11 tubular projections from endosomes, which were dynamic and remained elongated for several seconds (Figure 2b).

The distribution of Rab11 vesicles after CDKI-73 treatment was examined in relation to the plasma membrane using CellMask™ Deep Red. There were significantly more small (≤1 µm) Rab11 vesicles (*p* < 0.0001; Figure 2h) at the plasma membrane in controls (Figure 2c), compared to the fat body cells treated with CDKI-73 at the concentrations of 50 nM (Figure 2d), 500 nM (Figure 2e) and 1 µM (Figure 2f). The *lyst^RNAi^* depleted fat body cells also showed a reduced number of small (≤1 µm) Rab11 vesicles at the plasma membrane (Figure 2g), suggesting a similar effect to that observed for CDKI-73 treatment (Figure 2h).

### 3.3. CDKI-73 Depleted the Amount of Intracellular Drs During an Innate Immune Response

**Figure 3:**
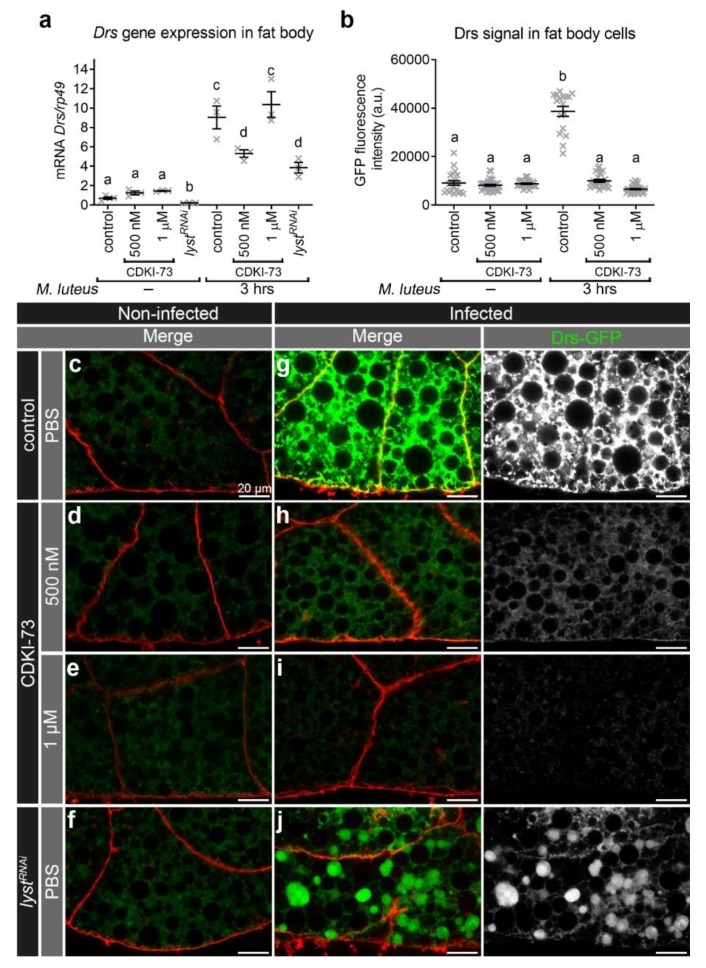
CDKI-73 depletes Drs in fat body cells after bacterial challenge. (**a**) The expression of *Drs* was characterised by qRT-PCR. (**b**) Histogram showing comparative analysis of intracellular Drs-GFP signal in fat body cells. One-way ANOVA and Tukey’s multiple comparison test showed significant differences between the means in designated groups (depicted by different letters on the bars in (**a**) and (**b**), *p* < 0.0001). Data are represented as mean ± SEM. (**c**–**j**) Confocal micrographs showing distribution of Drs-GFP (green) in fat body cells. The plasma membrane was outlined by CellMask™ Deep Red (red). Representative images were from fat body tissues treated for 30 min either with PBS (**c**,**f**,**g**,**j**), CDKI-73 at 500 nM (**d**, **h**) or 1 μM (**e**, **i**). Fat body tissues were from non-infected (**c**–**f**) and infected (*Micrococcus luteus*) larvae (**g**–**j**). Fat body tissues were from the following genotypes: *CG-CAL4 > UAS-Rab11-GFP/+* (**c**–**e**, **g**–**i**) and *UAS-lyst^RNAi^/+; CG-CAL4 > UAS-Rab11-GFP/+* (**f**, **j**). Scale bars: 20 μm.

Treatment of fat body tissues with CDKI-73 at 500 nM and 1 μM produced stronger effect on Rab11 endosomes (Figure 1), and therefore these two concentrations were used to determine the inhibitory effect of the compound on innate immune cargo secretion. Prior to infection, *Drs* gene expression was similar in control and fat body tissues treated for 30 min at room temperature with CDKI-73 (500 nM and 1 μM). In *lyst^RNAi^* fat body cells, *Drs* mRNA was significantly reduced (Figure 3a), when compared to control and CDKI-73 treatment. Under conditions of oral bacterial challenge, there was a significant up-regulation of *Drs* transcription in control, CDKI-73 treated (500 nM and 1 µM) and *lyst^RNAi^* fat body cells three hours after infection (Figure 3a). While there was an increase in *Drs* mRNA in response to infection in fat body cells (Figure 3a), there was significantly less Drs-GFP detected after CDKI-73 treatment, when compared to the infected controls (*p* < 0.0001; Figure 3b,c–j). Treatment with 1 µM CDKI-73 (Figure 3h) appeared to reduce the amount of intracellular Drs-GFP by more than that for 500 nM CDKI-73 (Figure 3i), but considering experimental variation, this reduction was not statistically significant (Figure 3b). In *lyst^RNAi^* depleted fat body cells, Drs-GFP was detected, but it had a restricted distribution, being concentrated in large intracellular compartments (Figure 3j).

To further define the effect of CDKI-73 on innate immune cargo delivery, the localisation of the antimicrobial peptide Drs-GFP was defined at the plasma membrane of fat body cells before and after infection (Figure S3a–h). Treatment of fat body tissues with 500 nM (Figure S3f) and 1 µM CDKI-73 (Figure S3g) reduced the amount of Drs-GFP at the cell surface, when compared to the infected controls (Figure S3e). There was also a reduced amount of Drs-GFP at the surface of fat body cells from *lyst^RNAi^* transgenic larvae (Figure S3h). Quantitation confirmed that there was a significant reduction in Drs-GFP signal at the cell surface after treatment with CDKI-73 at concentrations of 500 nM or 1 μM and *lyst^RNAi^* depletion, when compared to the infected control (*p* < 0.0001; Figure S3i).

### 3.4. CDKI-73 Modulated the Secretion of Pro-Inflammatory Cytokines

**Figure 4:**
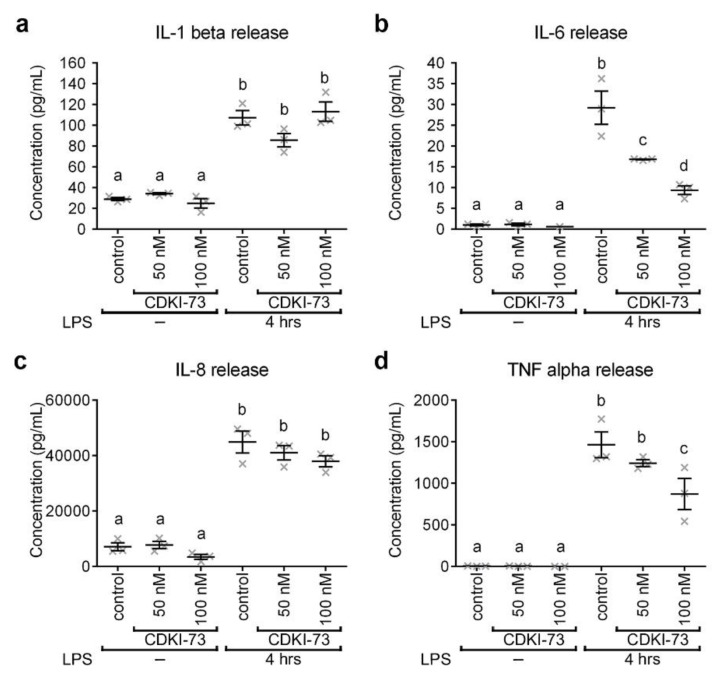
CDKI-73 reduces pro-inflammatory cytokine release by human THP-1 macrophages. (**a**–**d**) Comparative histograms showing the secretion of IL-1β (**a**), IL-6 (**b**), IL-8 (**c**) and TNFα (**d**) from lipopolysaccharide (LPS)-stimulated THP-1 macrophages after the addition of CDKI-73 compound. One-way ANOVA and Tukey’s multiple comparison test showed significant differences between the means in designated groups (depicted by different letters on the bars, *p* < 0.0001). Data are represented as mean ± SEM.

As it was not possible to determine secreted Drs-GFP in the insect hemolymph due to the extensive coagulation of this fluid, the effect of CDKI-73 treatment was studied on pro-inflammatory cytokines secreted by human THP-1 macrophages. Secretion of the four key proinflammatory cytokines (i.e., IL-1β, IL-6, IL-8 and TNFα), accountable for early stages of the immune response, were analysed before and after LPS-stimulation of the cells. In the absence of LPS stimulation, treatment with CDKI-73 at 50 nM and 100 nM had no discernible effect on the secretion of IL-1β (Figure 4a), IL-6 (Figure 4b), IL-8 (Figure 4c) and TNFα (Figure 4d), when compared to controls. The secretion of these cytokines was increased when macrophages were primed with LPS (Figure 4a–d). CDKI-73 did not result in a significant reduction in the amount of IL-1β detected in the supernatants of LPS-stimulated macrophages (Figure 4a). In contrast, IL-6 was reduced by 42–67%, when macrophages were treated with CDKI-73 (50 nM and 100 nM; *p* < 0.0001; Figure 4b). IL-8 secretion after LPS treatment was not significantly down-regulated by CDKI-73, when compared to controls (Figure 4c). The secretion of TNFα was significantly reduced in LPS-stimulated macrophages, when treated with 100 nM CDKI-73 (*p* < 0.05; Figure 4d).

**Figure 5:**
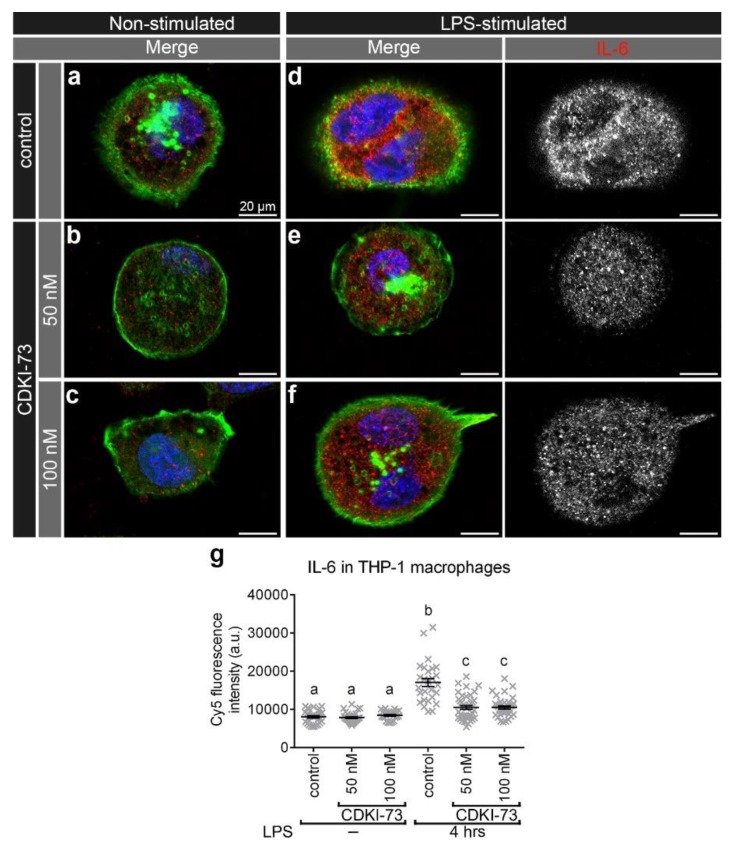
CDKI-73 depletes interleukin-6 (IL-6) in LPS-stimulated THP-1 macrophages. (**a**–**f**) Confocal micrographs showing IL-6 detected with anti-IL-6 antibody (red) in relation to the plasma membrane outlined with Alexa Fluor^®^ 488 Phalloidin (green). The nucleus was depicted by staining with Hoechst 33,258 DNA stain (blue). Representative images were from control THP-1 macrophages (**a**,**d**) and THP-1 macrophages treated either with CDKI-73 at 50 nM (**b**,**e**) or 100 nM (**c**,**f**) for four hours. The visualised macrophages were non-stimulated (**a**–**c**) and LPS-stimulated for six hours (**d**–**f**). Scale bars: 20 μm. (**g**) Histogram showing comparative analysis of IL-6-Cy5 signal in the designated samples. One-way ANOVA and Tukey’s multiple comparison test showed significant differences between the means in designated groups (depicted by different letters on the bars, *p* < 0.0001). Data are represented as mean ± SEM.

**Figure 6:**
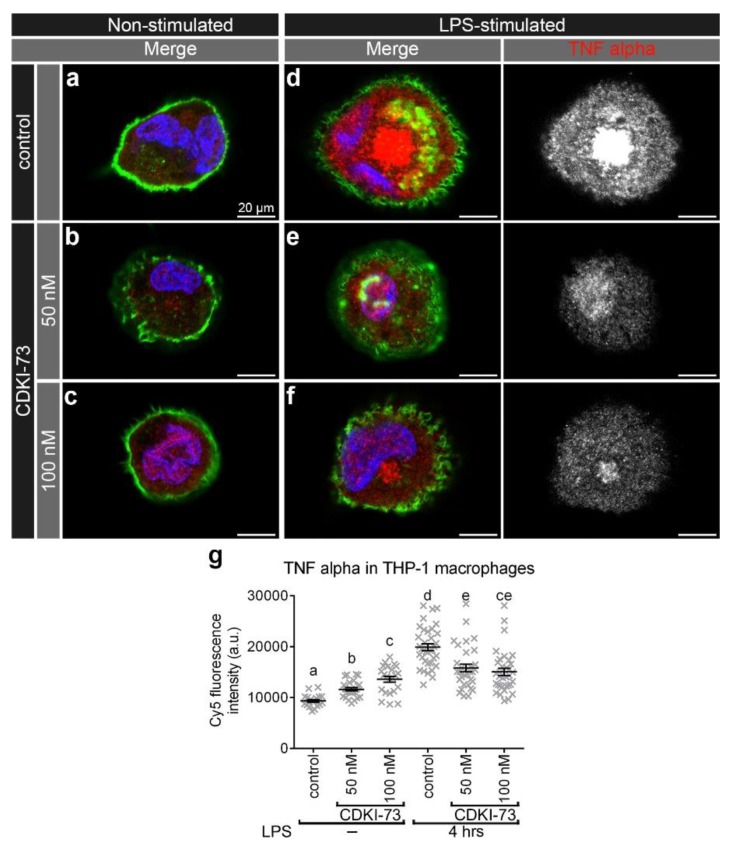
CDKI-73 depletes tumour necrosis factor alpha (TNFα) in LPS-stimulated THP-1 macrophages. (**a**–**f**) Confocal micrographs showing TNFα detected with anti-TNFα antibody (red) in relation to the plasma membrane outlined with Alexa Fluor^®^ 488 Phalloidin (green). The nucleus was depicted by staining with Hoechst 33,258 DNA stain (blue). Representative images were from control THP-1 macrophages (**a**,**d**), and THP-1 macrophages treated either with CDKI-73 at 50 nM (**b**,**e**) or 100 nM (**c**,**f**) for four hours. The visualised macrophages were non-stimulated (**a**–**c**) and LPS-stimulated for six hours (**d**–**f**). Scale bars: 20 μm. (**g**) Histogram showing comparative analysis of TNFα-Cy5 signal in the designated samples. One-way ANOVA and Tukey’s multiple comparison test showed significant differences between the means in designated groups (depicted by different letters on the bars, *p* < 0.0001). Data are represented as mean ± SEM.

To evaluate the effect of CDKI-73 treatment on the innate immune cargo, the intracellular distribution of IL-6 and TNFα was investigated in non-stimulated and LPS-stimulated THP-1 macrophages. The secreted levels of these two cytokines were significantly reduced, and so their intracellular distribution was further analysed in this study. Prior to LPS-stimulation, there was only a relatively small amount of IL-6 detected in control (Figure 5a) and CDKI-73 treated macrophages (Figure 5b,c). Upon stimulation with LPS, there were numerous IL-6 puncta with a widespread distribution throughout the cytoplasm of control macrophages (Figure 5d). CDKI-73 treatment resulted in a substantial reduction of IL-6 puncta (*p* < 0.0001; Figure 5e,f,g). While there was a higher IL-6 fluorescence intensity in CDKI-73 treated and LPS-stimulated macrophages, when compared to non-stimulated cells, this was significantly less than that detected in LPS-stimulated macrophages (Figure 5g). In addition, there was a limited amount of TNFα fluorescence in control and 50 nM–100 nM CDKI-73 treated macrophages (Figure 6a–c). Upon stimulation with LPS, there was a dramatic increase in TNFα fluorescence in control THP-1 macrophages (Figure 6d), but not in CDKI-73 treated cells (*p* < 0.0001; Figure 6e,f,g).

### 3.5. CDKI-73 Altered the Intracellular Distribution of Rab11 Vesicles in Human Macrophages

Pro-inflammatory cytokines can traffic through recycling endosomes and so their intracellular localisation was compared to Rab11 endosomes. This was examined in LPS-stimulated human THP-1 macrophages as the basal levels of pro-inflammatory cytokines in non-stimulated cells are low (Figure 4a–d, Figure 5a and Figure 6a). The pro-inflammatory cytokines IL-6 (Figure S4a) and TNFα (Figure S4b) appeared to be associated with Rab11 endosomes in LPS-stimulated THP-1 macrophages (Figure S4c).

**Figure 7:**
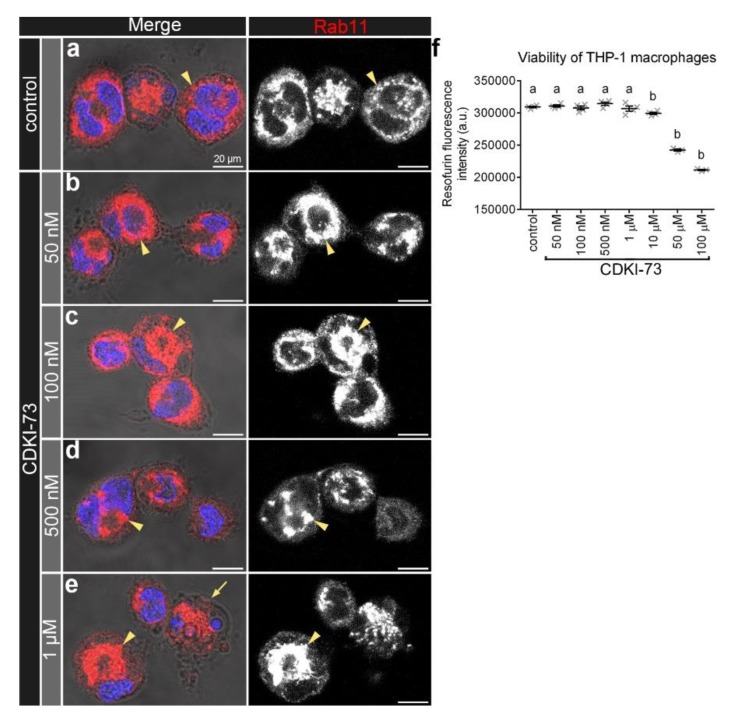
CDKI-73 causes accumulation of Rab11 endosomes in the perinuclear region of THP-1 macrophages. (**a**–**e**) Confocal micrographs showing Rab11 endosomes detected with anti-Rab11 antibody (red) in relation to the cell surface, detected by phase-contrast microscopy. The nucleus was depicted by staining with Hoechst 33,258 DNA stain (blue). Representative images were from control macrophages (**a**) and macrophages treated either with CDKI-73 at 50 nM CDKI-73 (**b**), 100 nM (**c**), 500 nM (**d**) or 1 μM (**e**) for four hours. Arrowheads depict Rab11 vesicles with altered distribution within the cell. Scale bars: 20 μm. (**f**) Histogram showing the levels of metabolic activity/redox state of macrophages after treatment with CDKI-73. One-way ANOVA and Dunnett’s multiple comparison test showed significant differences between the means in designated groups (depicted by different letters on the bars, *p* < 0.0001). Data are represented as mean ± SEM.

In control THP-1 macrophages (Figure 7a), Rab11 endosomes appeared to be less concentrated in the perinuclear region than in the same region of THP-1 macrophages treated with 50 nM–1 µM CDKI-73 (Figure 7b–e). Consequently, THP-1 macrophages treated with CDKI-73 had fewer Rab11 vesicles located near the cell surface (Figure 7d,e), when compared to controls (Figure 7a). Treatment with 1 µM CDKI-73 (Figure 7e) led to nuclear morphological changes, including nuclear fragmentation and chromatin condensation, when compared to both controls (Figure 7a) and lower concentrations of CDKI-73 (50 nM–500 nM; Figure 7b–d). However, treatment with CDKI-73 at 50 nM–1 µM did not appear to be cytotoxic as the viability of THP-1 macrophages was not significantly different from controls (Figure 7f). In contrast, at the higher concentrations of CDKI-73 (10 µM, 50 µM and 100 µM), the viability of THP-1 macrophages was significantly reduced (*p* < 0.0001; Figure 7f).

## 4. Discussion

Inflammatory disorders cause a significant burden to both patients and health care systems. Although fast-acting symptomatic drugs (non-steroidal anti-inflammatory drugs and glucocorticoids), slow-acting disease modifying anti-rheumatic drugs (methotrexate and cytostatic drugs) and immune-suppressants (calcineurin inhibitors, neutralisers of TNFα and IL-1) have provided some treatment options, patients often respond poorly to these drugs or the disease recurs once treatment has ceased. There is therefore a critical need to develop new therapeutics that enable effective control of the release of inflammatory immune mediators. Here, we have investigated a CDK inhibitor CDKI-73 for potential drug development to control the secretion of inflammatory cytokines.

Following infection with bacteria, the TLR4/MD-2 complex triggers the initiation of down-stream signalling events, resulting in the translocation of transcription factors from the cytoplasm into the nucleus [35]. For example, NF-κB and interferon regulatory factors can induce the transcription of multiple genes involved in antimicrobial defence, including antimicrobial peptides and pro-inflammatory cytokines [36,37]. The transcription factor NF-κB can interact with CDK9/cyclin T, the catalytic subunit of positive transcription elongation factor P-TEFb [38,39,40] and can be recruited to the IL-8 promoter in TNFα-treated cells [41]. In addition, the interaction of CDK9 with the IL-6 receptor suggests a role of CDK9 in signal transduction during an immune response [42,43]. Indeed, the CDK9 inhibitor flavopiridol can disrupt the transcriptional elongation of gamma fibrinogen gene without affecting STAT3 (signal transducer and activator of transcription 3) activation or complex formation with CDK9 [44]. In the current study, following treatment with CDKI-73, there was normal activation of NF-κB immune response pathways, resulting in gene expression of the antimicrobial peptide Drs, suggesting that this compound did not alter gene transcription. Given that CDKI-73 has previously been implicated in the control of eIF4E-mediated translation in cancer cells and had an effect on immune response [26], it was postulated that CDKI-73 might be acting on either translation, intracellular traffic or the secretion of immune mediators in *Drosophila* fat body tissues.

CDKI-73 decreases the phosphorylation of eIF4E by blocking MAPK-interacting kinase (Mnk) activity [26]. The reduced activity of eIF4E can selectively down-regulate the translation of mRNA encoding IκBα, an inhibitor of NF-κB [45]. Although reduced eIF4E phosphorylation causes enhanced production of interferon beta [45], in the present study the treatment with CDKI-73 reduced the amount of intracellular antimicrobial peptide Drs in *Drosophila* fat body tissues and the pro-inflammatory cytokines IL-6 and TNFα in LPS-stimulated THP-1 macrophages. This supported previous findings showing that reduced eIF4E phosphorylation has an impact on the translation efficiency of mRNAs, including mRNAs that encode TNFα, IL-6 and chemokines, in LPS-stimulated IL-1 receptor-associated kinase 2-deficient mice [46]. Although CDKI-73 treatment did not reduce the amount of secreted IL-1β by THP-1 macrophages, the treatment of LPS-stimulated macrophages with CDKI-73 led to a decrease in the secretion of IL-6 and TNFα. To further explore the effect of CDKI-73 treatment on immune response, future work needs to be focused on the analysis of a wider range of pro- and anti-inflammatory cytokines secreted by the macrophages. Moreover, future studies will be required to measure mRNA levels of these pro-inflammatory cytokines after CDKI-73 treatment.

Rab4/Rab11 recycling endosomes represent a critical point for cargo compartmentalisation and a potential point for divergence in the intracellular traffic of immune mediators [4,5]. The morphological changes to Rab11 endosomes caused by CDKI-73 treatment altered sorting and compartmentalisation of immune cargo in recycling endosomes, and therefore disrupted down-stream traffic in the secretory pathway. CDKI-73 treatment had no discernible inhibitory effect on the secretion of IL-1β and IL-8, and therefore their intracellular localisation needs to be determined in order to support a Rab11-dependent mechanism. In this study, CDKI-73 not only inhibited the delivery of Rab11 vesicles to the plasma membrane, but also resulted in the accumulation of large multivesicular Rab11 endosomes at the cell periphery. Interestingly, treatment of fat body tissues with CDKI-73 to some extent mimicked Chédiak-Higashi syndrome at the morphological level. Depletion of *Drosophila lyst* gene produced a similar vesicular phenotype, causing a reduced number of small Rab11 vesicles at the plasma membrane and accumulation of large multivesicular Rab11 endosomes in fat body cells. *lyst^RNAi^* also resulted in the accumulation of Drs cargo in these enlarged Rab11 endosomes, whereas CDKI-73 depleted the amount of Drs detected in immune cells. Despite the similarities, it is yet to be investigated whether these phenotypes are caused by similar alterations in endosomal dynamics. Given the normal level of mRNA, this suggested that CDKI-73 might be causing Drs immune cargo to be targeted for degradation, and this is yet to be determined using inhibitors. CDKI-73 caused an increase in the frequency of Rab11 endosome fusion events and this might have disrupted the formation of multivesicular Rab11 endosomes, altering immune sorting and directing cargo towards a lysosomal degradative pathway. The effects of CDKI-73 on endosome morphology did not appear to be due to direct targeting of CDK9, as the specific inhibitor DRB had a different effect, reducing the size of Rab11 endosomes. Future studies will be required to determine the mechanism of action, and possible off-targets interactions and toxicities of the CDKI-73 compound.

In addition to the effect on recycling endosome morphology, CDKI-73 impacted on Rab11 vesicle delivery towards the plasma membrane and reduced the secretion of antimicrobial peptide Drs, IL-6 and TNFα. CDKI-73 therefore appeared to have a dual role in the Mnk/eIF4E axis and endosomal trafficking pathways. We speculate that CDKI-73 may negatively regulate Rab4 and/or Rab11 activity. This would be consistent with our previous observations on *pkaap* depletion, which caused similar alterations in the endosomal morphology and Rab11-mediated trafficking [16]. Alternatively, CDKI-73 may indirectly target other key Rab proteins. Consequently, our findings highlight the need to further investigate the role of CDKI-73 in the regulation of innate immune secretion. Nevertheless, our study indicates that CDKI-73 not only limits cargo delivery, but also controls the amount of IL-6 and TNFα in the cells, giving rise to effective control over innate immune secretion.

## 5. Conclusions

CDKI-73 alters the secretion of the antimicrobial peptide Drs and pro-inflammatory cytokines IL-6 and TNFα. This not only involved reduced Rab11 vesicle delivery and cargo exocytosis from the plasma membrane, but also altered packaging and sorting in Rab11 recycling endosomes. The effect of CDKI-73 on Rab11 endosome morphology appeared to correlate with reduced amounts of antimicrobial peptide Drs, but not its gene expression, suggesting that the disruption to Rab11 endosomes might have directed this immune cargo for degradation. The dual effect of CDKI-73 on immune cargo packaging, sorting and Rab11 vesicle delivery to the plasma membrane provides a new way of regulating innate immune secretion to control inflammation.

## Supplementary Materials

The following are available online at https://www.mdpi.com/2073-4409/9/2/372/s1, Figure S1: CDKI-73 alters the number and morphology of Rab11 endosomes, Figure S2: CDKI-73 exhibits low cytotoxicity on fat body cells, Figure S2: CDKI-73 reduced amount of Drs at the cell surface, Figure S3: IL-6 and TNFα co-localise with Rab11 endosomes in LPS-stimulated THP-1 macrophages.
