# Supplementary material for: CDKI-73 Is a Novel Pharmacological Inhibitor of Rab11 Cargo Delivery and Innate Immune Secretion
Source: Cells. 2020 Feb 5;9(2):372. doi: 10.3390/cells9020372 (PMC7072129; doi:10.3390/cells9020372)
Supplement: Supplementary file 1 [file cells-09-00372-s001.pdf]

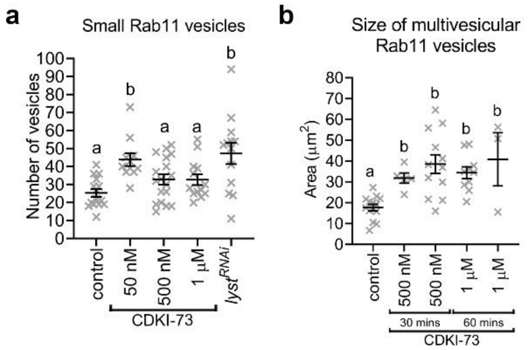

Figure S1. CDKI-73 alters the number and morphology of Rab11 endosomes. Histograms showing comparative analysis of the number of small ≤ 1 μm Rab11 vesicles throughout the fat body cells (**a**), and the size of multivesicular Rab11 endosomes within 30 and 60 minutes after CDKI-73 treatment (**b**). The analysis was performed within 40 μm^2 regions of interest. One-way ANOVA and Dunnett's multiple comparison test showed significant differences between the means in treatment groups (depicted by different letters on the bars, $p < 0.05$). Data are represented as mean ± SEM.

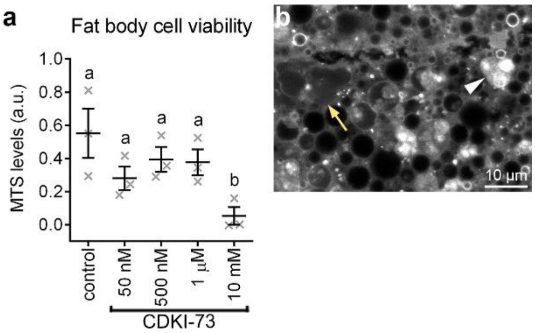

Figure S2. CDKI-73 exhibits low cytotoxicity on fat body cells. (**a**) Histogram showing comparative analysis of metabolic activity/redox state of fat body cells. One-way ANOVA and Dunnett's multiple comparison test showed significant differences between the means in treatment groups (depicted by different letters on the bars, $p < 0.0001$). Data are represented as mean ± SEM. (**b**) Micrograph of confocal cross-sections though the fat body cell showing altered structure of fat body cell (yellow arrow) upon treatment with 10 μM CDKI-73. Arrowhead depicts large multivesicular Rab11 endosomes. Scale bar: 10 μm.

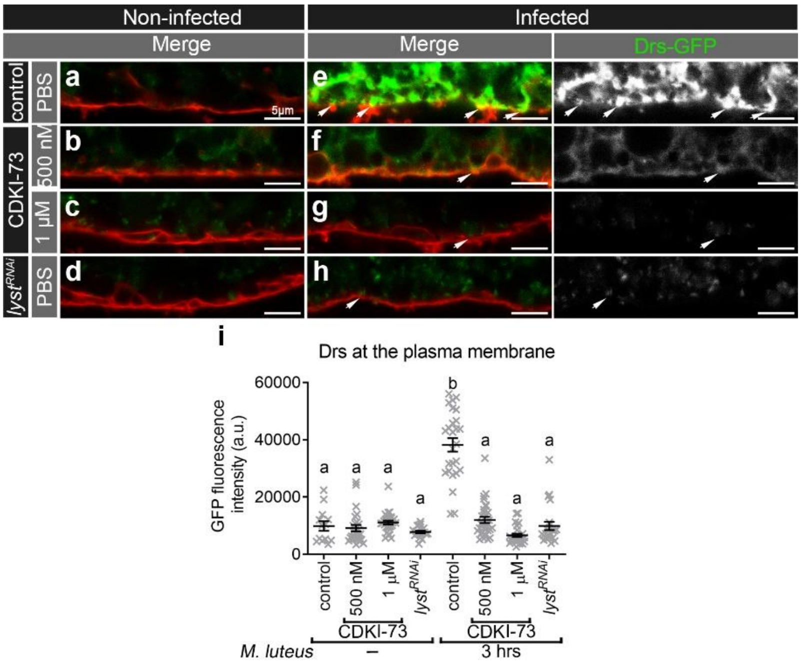

Figure S3. CDKI-73 reduces amount of Drs at the cell surface. (**a**-**h**) Confocal micrographs of cross-sections through the fat body cells showing Drs-GFP (green) in relation to the plasma membrane outlined by CellMask™ Deep Red (red). Representative images were from fat body tissues treated for 30 minutes either with PBS (**a**, **d**, **e**, **h**), CDKI-73 at 500 nM (**b**, **f**) or 1 μM (**c**, **g**). Fat body cells were visualised from non-infected (**a**-**d**) and infected (*Micrococcus luteus*) larvae (**e**-**h**). Fat body cells were from the following genotypes: *CG-CAL4* > *Rab11-GFP*/+ (**a**-**c**, **e**-**g**) and *UAS-lyst*^{RNAi}/+; *CG-CAL4* > *Rab11-GFP*/+ (**d**, **h**). Arrows depict Drs-GFP at the plasma membrane. Scale bars: 5 μm. (**i**) Histogram showing comparative analysis of Drs-GFP signal at the plasma membrane. One-way ANOVA and Tukey's multiple comparison test showed significant differences between the means in designated groups (depicted by different letters on the bars, $p < 0.0001$). Data are represented as mean ± SEM.

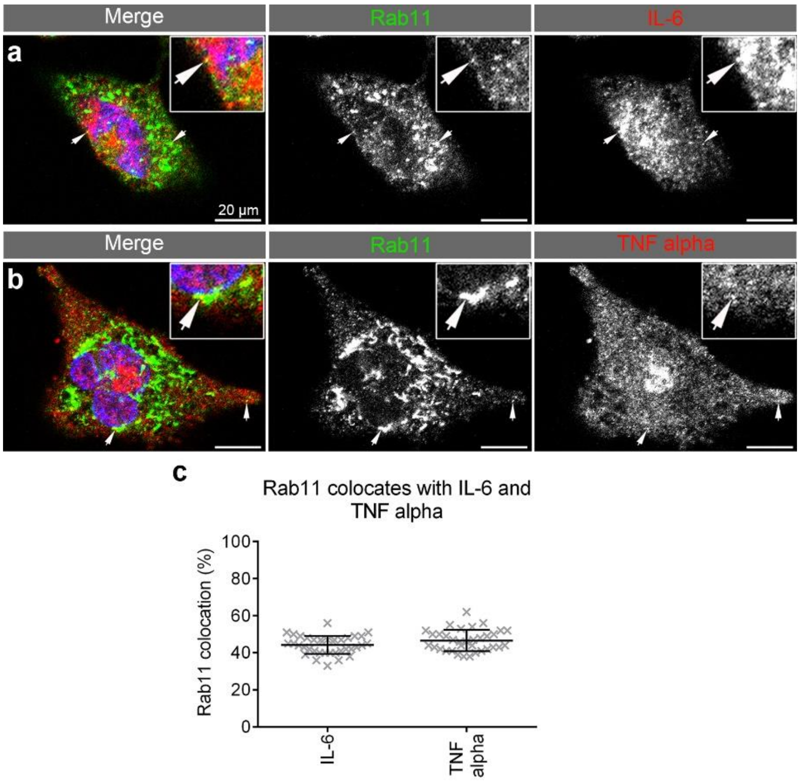

Figure S4. IL-6 and TNFα co-localise with Rab11 endosomes in LPS-stimulated THP-1 macrophages. (**a**, **b**) Confocal micrographs showing IL-6 (red in a) and TNFα (red in b) in relation to Rab11 endosomes (green in **a**, **b**). The nucleus was depicted by staining with Hoechst 33258 DNA stain (blue in **a**, **b**). Representative images were from LPS-stimulated THP-1 macrophages. Scale bars: 20 μm. (**c**) Percentage of Rab11 vesicles co-localisation with IL-6 and TNFα. Data are represented as mean ± SEM.
